# Better outcomes and reduced failures for arthroplasty over osteotomy for advanced compartmental knee osteoarthritis in patients older than 50 years

**DOI:** 10.1186/s13018-020-02079-6

**Published:** 2020-11-19

**Authors:** Filippo Migliorini, Arne Driessen, Francesco Oliva, Gayle D. Maffulli, Markus Tingart, Nicola Maffulli

**Affiliations:** 1 Department of Orthopaedic Surgery, RWTH University Hospital Aachen, Pauwelsstraße 30, 52074 Aachen, Germany; 2 Department of Medicine, Surgery and Dentistry, University of Salerno, Via S. Allende, 84081 Baronissi, SA Italy; 3 Wholelife Clinics, London, UK; 4 Centre for Sports and Exercise Medicine, Barts and the London School of Medicine and Dentistry, Queen Mary University of London, Mile End Hospital, 275 Bancroft Road, London, E1 4DG England, UK; 5 School of Pharmacy and Bioengineering, Keele University Faculty of Medicine, Thornburrow Drive, Stoke on Trent, England, UK

**Keywords:** Compartmental knee osteoarthritis, Unicompartmental knee arthroplasty, Open-wedge tibial osteotomy

## Abstract

**Background:**

Both compartmental knee arthroplasty (CKA) and open-wedge high tibial osteotomy (HTO) may be used to treat patients older than 50 years of age with advanced compartmental osteoarthritis (OA) secondary to leg axis deformities. A meta-analysis was conducted to clarify the role of open-wedge HTO versus CKA for patients older than 50 years with advanced compartmental knee OA. The present investigation aimed to analyse the clinical outcomes, implant failure and survivorship.

**Methods:**

This meta-analysis was performed in accordance with the PRISMA guidelines. In September 2020, the main online databases were accessed. All clinical trials comparing the outcomes of open-wedge HTO versus CKA for compartmental knee OA were considered. Data analysis was performed through the Review Manager Software 5.3 (the Nordic Cochrane Collaboration, Copenhagen). Implant survivorship was analysed with a Kaplan-Meier (KM) curve that was performed using the STATA/MP software (StataCorp, College Station, TX).

**Results:**

Data from 618 (HTO, 307; CKA, 311) patients were collected. Good baseline comparability among patient age, BMI and gender was detected. The Tegner Activity Scale was higher in the CKA group (*P* = 0.04), as were the Lysholm score (*P* = 0.001), the International Knee Documentation Committee (*P* = 0.0001) and the Knee injury and Osteoarthritis Outcome Score (*P* = 0.05). At a mean follow-up of 5 years, revisions were less in the CKA cohort (OR, 2.27; *P* = 0.004). The Kaplan-Meier curve evidenced longer implant survivorship in favour of the CKA group (*P* = 0.01).

**Conclusion:**

In patients older than 50 years of age with compartmental knee OA secondary to frontal axis leg deformities, CKA performed better than open-wedge HTO.

## Introduction

Degenerative joint disease, such as osteoarthritis (OA) in the knee, is a common cause of complaint in older patients and carries an increasing financial burden [1]. Although knee OA can affect all three compartments of the knee, up to 30% of patients have evidence of only single compartmental degeneration [2, 3]. Despite multifactorial occurrences, compartmental knee OA is often caused by a deviation of the mechanical axis of the knee [4, 5]. Therefore, unloading the affected compartment may decelerate deterioration of the osteoarthritic joint. Open-wedge high tibial osteotomy (HTO) is considered a suitable surgical option for patients with compartmental OA secondary to mechanical axis deviation [6–8]. In the last decades, however, there has been a growing interest in unicompartmental knee arthroplasty (CKA), which achieves excellent results along with very low rates of complications in single compartmental OA [9–12]. Traditionally, the open-wedge HTO was regarded to be the procedure of choice for active young (male) patients with preserved joint stability, preserved range of motion and absence of bi- or tricompartmental OA [13–15]. In contrast, a CKA was elected for older and less active patients with preserved range of motion, ligament stability and compartmental knee OA [16, 17]. Today, these indications have become less well defined, particularly in patients older than 50 years of age. Improvements in surgical techniques, implants and instruments, along with similar indications for both procedures, complicate the decision-making process [18]. Many authors agree that both HTOs and CKAs are valuable solutions for compartmental knee OA [13, 19, 20]. Thus, although CKA and HTO are established methods for surgical treatment of compartmental OA, the optimal intervention remains controversial. In the literature, a lack of evidence concerning the role of HTO versus CKA for compartmental knee OA exists [21–23]. Controversial results have been reported and are therefore currently debated [24–26]. Hence, a meta-analysis of clinical trials was conducted to clarify the role of open-wedge HTO versus CKA for compartmental knee OA. The present investigation aimed to analyse the clinical outcomes, failure and survivorship among the two surgical techniques.

## Material and methods

This meta-analysis was performed according to the Preferred Reporting Items for Systematic Review and Meta-Analysis: the PRISMA guidelines [27]. The search strategy was performed in accordance with the following criteria:

- P (population): Compartmental knee OA
- I (intervention): Open-wedge proximal tibial osteotomy
- C (comparison): Unicompartmental knee arthroplasty
- O (outcomes): Clinical scores, surgical revision and implant survivorship

### Literature search

Two independent reviewers performed the literature search (FM; AD). In September 2020, the main databases were accessed: Pubmed, Google Scholar, Embase and Scopus. For the search, the following keywords were combined: *knee*, *unicompartmental*, *unicondylar*, *compartmental*, *partial*, *arthrosis*, *degeneration*, *medial*, *arthroplasty*, *replacement*, *CKA*, *osteotomy*, *tibial*, *open wedge*, *Lysholm*, *Tegner*, *IKDC*, *KOOS*, *revision*, *survivorship*, *total knee arthroplasty*, *TKA*, *failure*, *pain*, *surgery*. The same authors performed the data extraction. First, the titles were screened and if they were considered to be of interest, the abstract was screened as well. Secondly, the full-text version of these articles was accessed. The bibliographies of the articles of interest were examined as well.

### Eligibility criteria

All clinical trials comparing the outcomes of open-wedge HTO versus CKA for compartmental knee OA were considered. Articles with levels of evidence I to III, according to the Oxford Centre of Evidenced-Based Medicine [28], were included. Given the author’s language capabilities, articles in English, Italian, German, French and Spanish were considered for inclusion. Registry studies were excluded. Case series and/or case reports, reviews, systematic reviews and meta-analyses were excluded. Given the rapid advancement and optimization of prosthetic implants and surgical techniques, only studies published after the year 2000 were considered for inclusion. Only articles reporting data on patients older than 50 years of age were considered for inclusion. Only articles reporting data on advanced knee joint degeneration (grade III to IV according to Kellgren and Lawrence score systems [29]) were eligible. Only articles reporting quantitative data under the outcomes of interest were included. Missing data under the preferred endpoints warranted exclusion from the present study. Disagreements between the authors were mutually debated and resolved by a third author (MT).

### Outcomes of interest

Two independent authors (FM; AD) extracted data from the articles of interest, including name of the authors, year of publication, type of study, number of knees, female/male ratio, mean age, BMI and type of implant. The outcomes of interest were the analysis of the clinical and functional scores (Lysholm Knee Scoring Scale [30], Tegner Activity Scale [31], International Knee Documentation Committee (IKDC) [32], Knee injury and Osteoarthritis Outcome Score (KOOS) [33]). Moreover, the rate of the failure (revision to total knee arthroplasty) and implant survivorship were also investigated.

### Methodological quality assessment

Methodological quality assessment was performed by two authors (FM; AD). For the methodological quality assessment, the risk-of-bias summary tool Review Manager Software 5.3 (the Nordic Cochrane Collaboration, Copenhagen) was used. The following biases were analysed: randomization and allocation bias (selection bias), blinding (detection bias), incomplete data (attrition bias), selective reporting (reporting bias) and other bias.

### Statistical analysis

The statistical analyses were performed by the main author (FM). To analyse baseline comparability among the samples, the unpaired *t* test was used, with values of *P* > 0.5 considered satisfactory. Data comparisons were performed with Review Manager Software 5.3 (the Nordic Cochrane Collaboration, Copenhagen). For continuous data, the inverse variance method was adopted. The mean difference was adopted as effect measure. For dichotomic data, the Mantel-Haenszel method was considered. The odd ratio (OR) was used to evaluate the effect measure. As for the analysis model, a fixed effect was chosen. The Chi-squared (*χ*^2^) and Higgins tests (*I*^2^) assessed heterogeneity. If *χ*^2^ > 0.5, the *I*^2^ test was performed. *I*^2^ test values of 25%, 50% and 75% detected low, moderate and high levels of heterogeneity, respectively. If high heterogeneity was detected, a random effect model was used. To assess the implant survivorship (revision to total knee arthroplasty), the Kaplan-Meier (KM) curve was performed using the STATA/MP software version 16.1 (StataCorp, College Station, TX). The KM curve was performed according to the Cox-regression through the Breslow method by hazard ratio (HR) effect measure. The confidence interval (CI) was set to 95% in all analyses. A *P* value < 0.05 was considered statistically significant.

## Results

### Search result

**Fig. 1:**
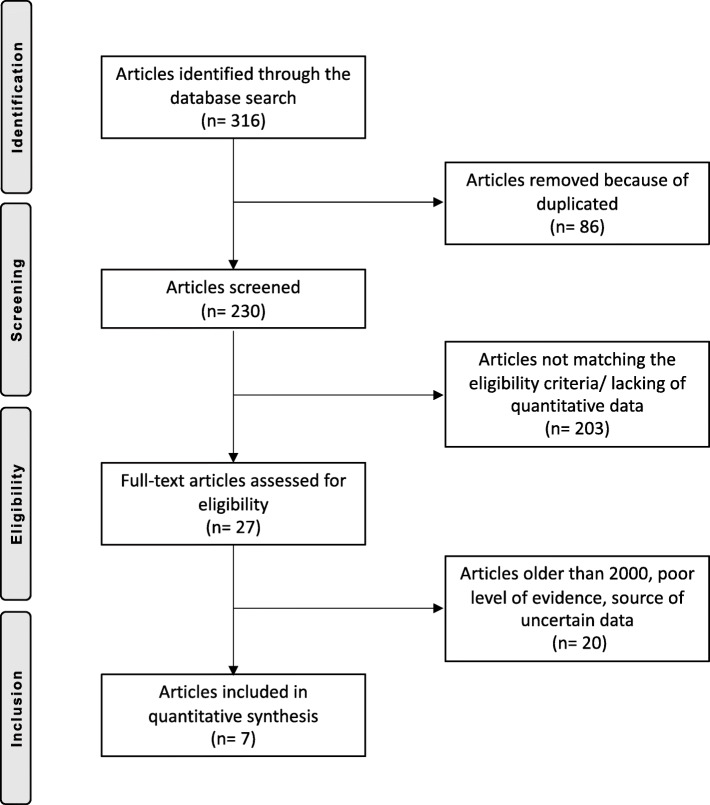
Flow chart of the literature search

A total of 316 articles were identified during the initial search, of which 86 were redundant and, therefore, excluded from the study. A further 203 articles were excluded due to not reporting data under the outcomes of interest or not matching the eligibility criteria. This left 27 studies for inclusion. Another 11 articles were excluded because they were published before the year 2000. A further seven studies were rejected due to poor level of evidence. Two articles were excluded being based on data of unreliable sources. Three articles reported data from national registries and therefore were not eligible for inclusion. This left a total of seven studies for analysis. The flow chart of the literature search is illustrated in Fig. 1.

### Risk of publication bias

**Fig. 2:**
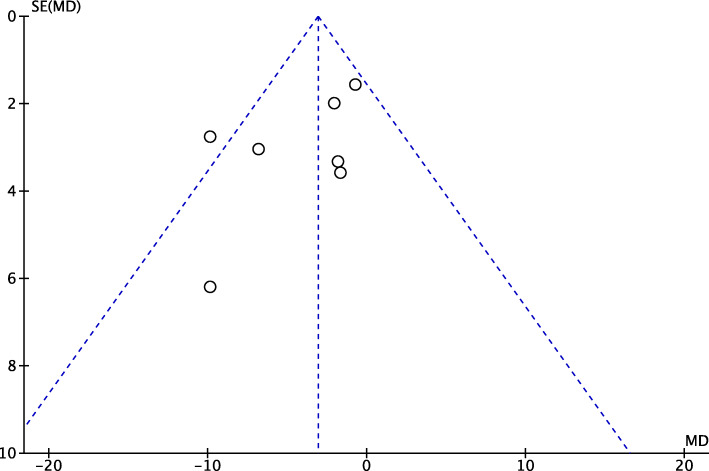
Funnel plot of the outcome Lysholm score

A funnel plot was generated to assess the risk of publication bias. We referred to the most commonly reported outcomes for analyses (Lysholm score). The analysis of the funnel plot detected a good distribution of the referral points. Most of the referral points were detected close to the confidence of acceptability. The funnel plot is shown in Fig. 2.

### Methodological quality assessment

**Fig. 3:**
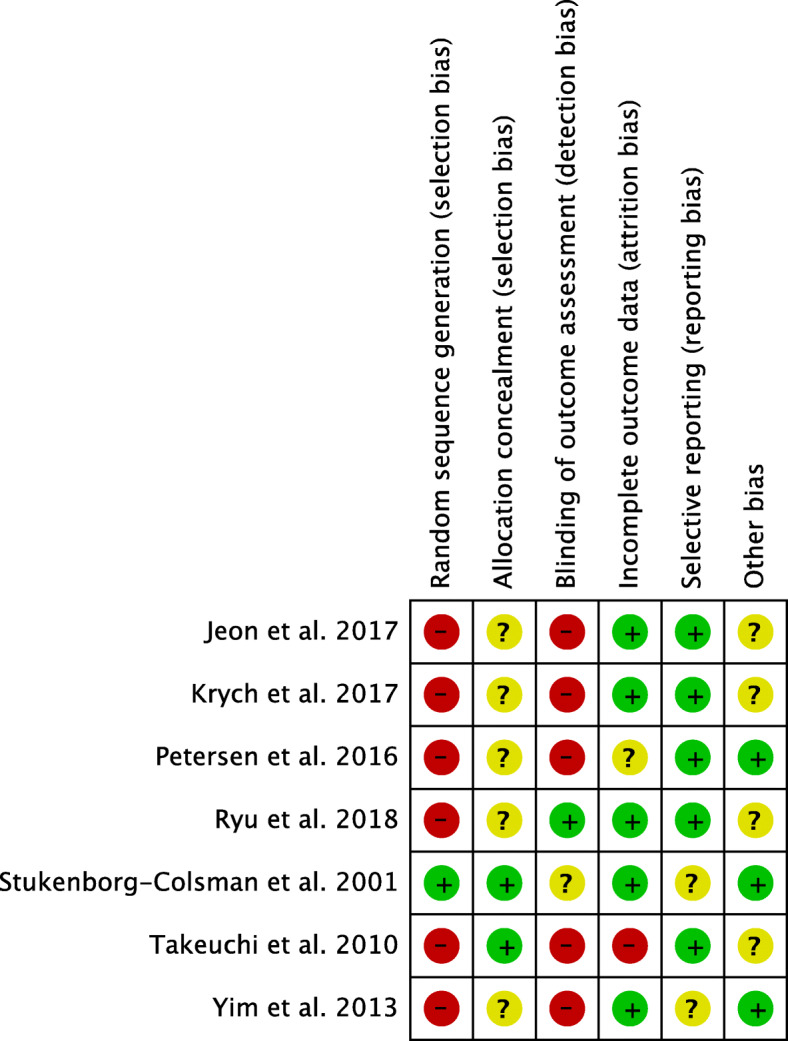
Authors’ judgements regarding each risk of bias for included studies

The quality of the methodological assessment scored fair to moderate. The reduced overall quality of the included studies negatively influenced the evidences concerning the present study. Most of the studies are retrospective, representing an important limitation. Authors’ judgements regarding each risk of bias item for each included study is shown in Fig. 3.

### Patient demographic

Data from 618 patients were included. The mean follow-up was 58.69 ± 24.9 months. In the HTO group, a total of 307 patients were collected. 34.5% (106 of 307 patients) were women. The mean age was 56.1 ± 6.6 years, the mean BMI was 28.7 ± 6.6 kg/m^2^. A total of 311 patients were included for analysis in the CKA group. 43.1% (134 of 311 patients) were women. The mean age was 57.8 ± 9.6 years, and the mean BMI was 28.7 ± 3.5 kg/m^2^. The unpaired *t* test detected a good baseline comparability among patient age, BMI and gender between the two cohorts (*P* = 0.5, *P* = 0.9 and *P* = 0.5, respectively). Patient demographics are shown in Table 1.

**Table 1:** Generalities and demographic data of included studies

Author, year	Type of study	Follow-up (months)	Type of procedure	Number of knees	Female (%)	Mean age	Mean BMI	Type of implant
Jeon et al., 2017 [34]	RCS	24	OWHTO	26	84.60	56.8	26.6	TomoFix (DePuy Synthes)
			CKA	21	80.90	60.7	26.1	High Flex (Zimmer)
Krych et al., 2017 [35]	RCS	84.00	OWHTO	30	28.30	48.0	31.9	Not specified
			CKA	133	55.20	47.0	32.5	Miller-Galante (Zimmer)
Petersen et al., 2016 [36]	RCS	60	OWHTO	13	39.10	58.9	23.0	TomoFix (DePuy Synthes)
			CKA	25	64.00	60.7	25.0	Oxford III implant
Ryu et al., 2018 [37]	RCS	36.55	OWHTO	23	91.30	57.6	27.7	ChronOS vivify spacer + TomoFix (DePuy Synthes)
			CKA	22	86.50	60.5	25.4	Sigma unicompartmental knee (DePuy)
Stukenborg-Colsman et al., 2001 [38]	RCT	90	OWHTO	32	40.60	67.0		Five-hole-two-thirds tubular plate and a cortical screw
			CKA	30	78.60	67.0		Unicondylar knee sliding (Aesculap)
Takeuchi et al., 2010 [39]	RCS	72.5	OWHTO	27	75.00	67.0		TomoFix (DePuy Synthes)
			CKA	30	47.10	77.0		Uni-Knee (Nakashima Propeller Co)
Yim et al., 2013 [40]	RCS	43.8	OWHTO	58	96.00	58.3		2 wedge plates (Aesculap)
			CKA	50	87.90	60.3		Miller-Galante (Zimmer)

*RCS* retrospective clinical study, *RCT* randomized clinical trial

### Functional and clinical outcomes

The Tegner activity scale scored greater in favour of the CKA group (EE, 0.69; 95% CI, 1.35 to 0.03; *P* = 0.04), as well as the Lysholm score (EE, 3.07; 95% CI, 4.95 to 1.19; *P* = 0.001), the IKDC (EE, 8.89; 95% CI, 13.48 to 4.29; *P* = 0.0001) and the KOOS (EE, 2.27; 95% CI, 8.77 to − 4.23; *P* = 0.05). A synopsis of these results is shown in Table 2.

**Table 2:** Synopsis of scores and data extracted from studies eligible for analysis

Outcome	Procedures (***n***)	Estimated effect [95% CI]	***I***^**2**^ (%)	***P***
Tegner	418	0.69 [1.35 to 0.03]	72	0.04
Lysholm	616	3.07 [4.95 to 1.19]	48	0.001
IKDC	141	8.89 [13.48 to 4.29]	0	0.0001
KOOS	141	2.27 [8.77 to − 4.23]	0	0.5
Revision	218	2.27 [0.50 to 10.34]	68	0.004

### Failure rate

**Fig. 4:**
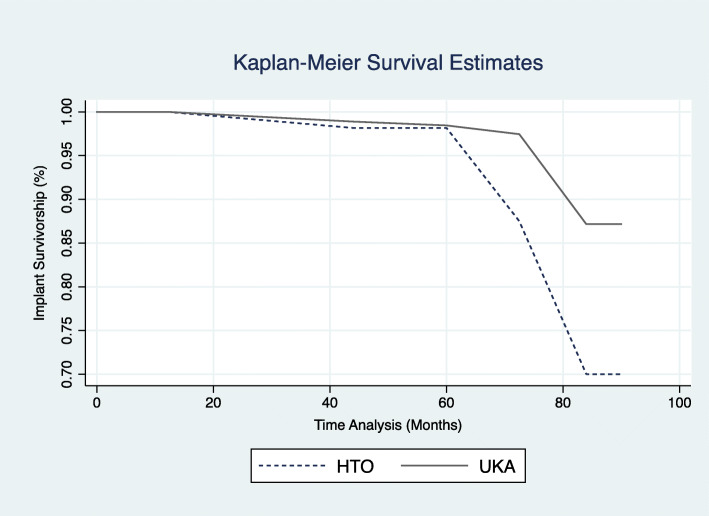
Kaplan-Meier curve

A reduced rate of surgical failure was noted in the CKA group (OR, 2.27; 95% CI, 0.50 to 10.34; *P* = 0.004; Table 2). These observations were even evidenced by the Kaplan-Meier curve (Fig. 4). The analysis was performed on a total of 484 observations (49 failures), resulting in a statistically significant (*P* = 0.01) longer survivorship in favour of the CKA group.

## Discussion

According to the main findings of this meta-analysis, CKA performed better overall. In patients older than 50 years with compartmental knee OA secondary to frontal leg malalignment, the CKA group achieved a statistically significant greater Tegner Activity score, Lysholm score, IKDC and KOOS, along with a statistically significant lower rate of revision surgeries compared to open-wedge HTO. This last point was also verified by the Kaplan-Meier curve at a mean of 5 years follow-up.

Lower limb frontal plane alignment is a determining factor for knee load distribution, and malalignments are a common cause of OA [26, 27]. According to Bellemans et al. [28], around 30% of males and 17% of females in Europe present with malalignment of the frontal plane > 3°. HTOs unload the involved compartment, transferring the load towards the centre or to the lateral compartment as evidenced in biomechanical studies [29]. Load transfer to the lateral compartment reduces joint pain hence improving knee function [26]. Studies combining mechanical readjustment with arthroscopic procedures (e.g. debridement, synovectomy or microfracture), or soft tissue transplantation/regeneration techniques, could improve outcomes compared to an isolated HTO [41, 42]. However, alignment corrections through HTO failed to ameliorate compartment degeneration. It has been stated that the HTO delays total joint replacement for roughly 10 years, with accurate corrections [26, 43, 44]. Concerning the CKA, axial correction of malalignment is infrequent. However, in the present study, CKAs resulted in a better clinical function and greater patient activity scores, reducing pain and stiffness and the need for revision surgeries, in addition to improving implant survivorship. Another limitation of HTOs are long rehabilitation and weight bearing restrictions for a minimum of 6 weeks (or longer), which may contribute to less desirable results [45, 46]. Especially for older patients, this may represent an important functional limitation and risk of residual immobilization [19]. In a recent meta-analysis of 13,789 patients [47], CKA was an effective solution for the treatment of end-stage compartmental knee degeneration, presenting a low complication rate, short surgical duration, low estimated blood loss and short hospitalization time. Compared to HTO, CKA patients presented increased maximum walking speed and single stance phase at 6 months follow-up [25, 48]. In their epidemiological study with data derived from a US private practice database, Nwachukwu et al. [49] evidenced an increasing trend of CKA at the expense of HTO for compartmental knee OA. Schindler et al. [50] found similar results in the UK.

Current evidence on the topic is controversial, and results from latest meta-analyses are contrasting. Fu et al. [22] performed a meta-analysis on 5840 knees (CKA 5081, HTO 759). Santoso et al. [21] performed a meta-analysis on 6538 knees (CKA 5497, HTO 1041), while Cao et al. [3] on 6222 knees (CKA 5335, 887 HTO). In contrast to the present study, they included both open- and closed-wedge HTOs, national registry data and studies in a timeframe between 1982 and 2017. Fu et al. [22] found no differences in revision and complication rate between the groups. However, Cao et al. [9] evidenced greater revision-rate and probability of complications in the HTO group. Santoso et al. [21] found no difference in revision surgeries but more complications in the HTO group. The results of knee-related functional scores are controversial. According to the current evidence, all studies agree that the HTO promotes better range of motion and no influence on progression of OA. Results of the present study demonstrate superior outcomes of CKA in all factors analysed.

The present meta-analysis has several limitations. Current evidence lacks high-quality studies, sample randomization and blinding methods on the topic. Inclusion and exclusion criteria were not analysed in the present investigation, representing another important limitation. The overall allocation process was unclear in most studies, leading to high risk of selection bias. Some authors discuss with their patients about the possible advantages and contraindications after informed consent, and the decision whether CKA or HTO was taken by mutual agreement is unclear [36, 37, 40]. Given these limitations, data from the present study must be interpreted with caution. Strengths of this work are the comprehensive nature of the literature search along with the strict eligibility criteria. Moreover, an optimal baseline comparability among patients age, BMI and gender was detected. Further studies should improve the evidence regarding the treatment of advanced compartmental knee OA, collecting more patients and improving the quality of recommendations through sample randomization and blinding.

## Conclusion

In patients older than 50 years with compartmental knee OA secondary to frontal leg malalignments, CKA achieved a statistically significant greater Tegner Activity Score, Lysholm score, IKDC and KOOS, along with a statistically significant lower rate of revision surgeries compared to open-wedge HTO. This last point was also verified by the Kaplan-Meier survival curve at a mean of 5 years follow-up.

## Data Availability

This study does not contain any third material.

## Abbreviations

CKA: Compartmental knee arthroplasty
HTO: Open-wedge high tibial osteotomy
OA: Osteoarthritis
KM: Kaplan-Meier
HR: Hazard ratio
IKDC: International Knee Documentation Committee
KOOS: Knee injury and Osteoarthritis Outcome Score
